# Effect of Addition of Antifungal Agents on Physical and Biological Properties of a Tissue Conditioner: An In-Vitro Study

**DOI:** 10.15171/apb.2017.059

**Published:** 2017-09-25

**Authors:** Pragati Rawat, Swatantra Agarwal, Siddhi Tripathi

**Affiliations:** Department of Prosthodontics and Crown & Bridge, Kothiwal dental college and research center, Moradabad, UP-244001, India.

**Keywords:** Candida albicans, Viscoelasticity, Tensile bond strength, Fluconazole, Oregano oil, Virgin coconut oil

## Abstract

***Purpose:*** Tissue conditioners are used for healing of abused oral tissues. They may harbour microorganisms causing oral diseases such as candidiasis compromising the health of the patient. Also, addition of antifungal agents into tissue conditioner may alter its properties. This study compares the anti-fungal property and mechanical properties of tissue conditioner containing different antifungal agents.

***Methods:*** Three antifungal agents, one synthetic – fluconazole, and two natural - oregano oil and virgin coconut oil were added into the tissue conditioner (Viscogel) in different concentrations. The antifungal property, tensile bond strength and viscoelasticity of Viscogel containing these antifungal agents were assessed after 24 hours, three days and seven days.

***Results:*** While, the highest antifungal activity was shown by Viscogel containing fluconazole, the maximum tensile bond strength was found to be of Viscogel alone (control). Although Viscogel alone and in combination of fluconazole showed deterioration in viscoelasticity, Viscogel in combination of natural agents showed no significant changes over the period of seven days.

***Conclusion:*** Incorporation of the natural agents in the tissue conditioner can be used as an effective alternative to systemic or topical synthetic antifungal agents.

## Introduction

A removable dental prosthesis is one of the commonest mode of treatment in prosthetic dentistry. However, their long term use without rest to underlying oral tissues may lead to adverse effect on their integrity. Both rest and tissue conditioners have been advocated to allow the deformed tissues of the residual ridges to return to normal form. The tissue conditioners are soft, resilient temporary liners that have been widely used in dentistry to manage multitude of patient problems and for various clinical applications.^1^ Apart from reducing and evenly distributing stresses on the mucosa of the basal seat^2^ they can also be used for temporary relining of immediate dentures etc.^3^ However, microbial growth results from the adherence of microbial cells promoted by rough surface, and from adhesive interactions between Candida species and oral bacteria, mostly Candida albicans that may lead to Candidiasis.^4^

The treatment of Candidiasis includes denture repair or replacement, adoption of prophylactic measures by the patient and the prescription of topical and systemic antifungal drugs.^5^ However, the success of topical application of drugs in the oral cavity may be compromised by the copious flow of saliva as well as by the lack of patient compliance. Therefore, antifungal agents can be incorporated in tissue conditioners to simultaneous treat injured peri-prosthetic tissues and infection by Candida.^6^ More recently, azole antifungal compounds such as fluconazole, which have excellent efficacy-toxicity profiles, have emerged as the principal drugs used in the treatment of candida infections. However, fluconazole produces few side effects: nausea, vomiting and might lead to the development of drug resistance in Candida albicans.^7,8^ So, to overcome these unpleasant side effects of chemically synthetic antifungals, herbs with antimicrobial property, especially anti-candida effect are of interest.^9,10^ Out of the various natural essential oils such as Oregano oil,^11^ Virgin coconut oil^12,13^ Melaleuca alternifolia oil,^14^ etc incorporated by different researchers in tissue conditioner, origanum oil and coconut oil have been found to exhibit superior antifungal activity. However, their use in dental applications is needed to be investigated more.

The most common problems encountered using soft denture liners are water sorption and solubility which are associated with Candida albicans growth and stresses at the liner/denture base interface leading to reduction in the bond strength.^15^ However, no data is available to ascertain the effect of incorporation of fluconazole and virgin coconut oil in the tissue conditioner on it’s tensile bond strength to denture base.

Also, the viscoelastic properties of soft denture liners are important since they characterize the ability of the material to exert a cushioning effect on the oral tissues and maintain shape during function.^16^ The temporary nature of these types of materials is because of the leaching of the alcohol and the plasticizer and adsorption of water and saliva which causes loss of viscoelasticity and therefore, compliance.^17^ Apart from few studies, there are no studies to comment upon the effect of incorporation of fluconazole, oregano oil and virgin coconut oil in the tissue conditioner on it’s viscoelastic properties.

Although, Virgin coconut oil and Oregano oil has been found to provide good anti-fungal protection, scarce investigations has been done for their use in dental applications. Moreover, no conclusive data is available for comparison between anti-fungal property, tensile bond strength and viscoelasticity of tissue conditioners when incorporated with oregano oil, virgin coconut oil and fluconazole. Accordingly, this study was designed to compare and evaluate the aforementioned parameters.

## Materials and Methods

Test materials used for the study were divided into following four groups having 10 samples each :

1. Group I - Visco Gel without any antifungal agents (control)
2. Group II - Visco Gel + 10 %w/w Fluconazole
3. Group III - Visco Gel + 60% v/v Oregano Oil
4. Group IV - Visco Gel + 25% v/v Virgin Coconut oil

Following methodology was employed for the study:

### Antifungal property

Candida albicans strain (ATCC NO. 24433) was taken and inoculated in Sabouraud dextrose agar broth which was incubated at 37°C for 24 hours. Standardization was done by diluting with sterile broth to a density visually equivalent to barium sulphate, standard Mc farland tube no. 5. It was then dispensed into a sterile Sabouraud agar plate and was allowed to dry. When the petri dishes had dried, four wells of diameter 6 mm and depth 1 mm were punched using a tissue punch on each of the ten petri dishes such that the wells lie within the lawn culture. Each of the four wells on each petri dish was filled with different test materials. After the materials had set in the wells, the plates were incubated at 37°C. The plates were then taken out after 24 hours, 3 days and 7 days and on each day the Minimum Inhibition Zone (MIZ) was measured using a metallic scale and a divider.^11^ Two diametric readings were taken for each sample and their mean was taken as the final reading for that sample

### Tensile Bond Strength

For fabrication of test samples, two wax blocks of slightly larger dimensions i.e. 41 mm x 11 mm x 11 mm each were made. Out of the two, one had 3 mm raise borders. These samples were polymerized with heat cure denture base resin using long curing cycle at 74°C for eight hours. The blocks that were obtained were finished and polished. These finished blocks were used to make silicone rubber moulds. These moulds were then used to make 40 more wax blocks which were polymerized and finished in the similar manner to the required dimensions i.e. 40 mm x 10 mm x 10 mm. One side of each block that was having raised border was roughened using 100-grit sandpaper and acrylic trimmer. Test materials in suitable consistency were applied on the side of the block which was roughened then on top of it another block without the raised border was placed as shown in. After setting, the excess material was trimmed and samples were stored in 100 ml distilled water at 37°C.^11^ Tensile bond strength was evaluated on Universal Testing Machine (Banbros Engineering Pvt. Ltd., Model no.-WDW-5) at a cross-head speed of 5 mm/min after 24 hours, three days and seven days.

### Viscoelasticity

A total of 40 dumbbell shaped moulds measuring 50 mm x 10 mm x 10 mm were prepared using Hydraulic Press. These moulds were placed over a metal platform before the test materials were poured into them. After the material had set, the dumbbell shaped samples were retrieved from the moulds and stored in 100 ml distilled water at 37°C. These specimens were then subjected to stress relaxation tests on Universal Testing Machine (Dak Test Bench, Samruddhi Comercial complex, Model no.- UTB 9052) at a crosshead speed of 20 inches/minute after 24 hours, three days and seven days and a load-deformation curve along with the elastic modulus was obtained for each specimen.^18^

### Statistical Analysis

The statistical analysis was done using SPSS (Statistical Package for Social Sciences) Version 15.0 statistical Analysis Software in which the obtained data was subjected to ANOVA and Post-Hoc Tests (Tukey-HSD).

## Results

### Antifungal property

**Figure 1:**
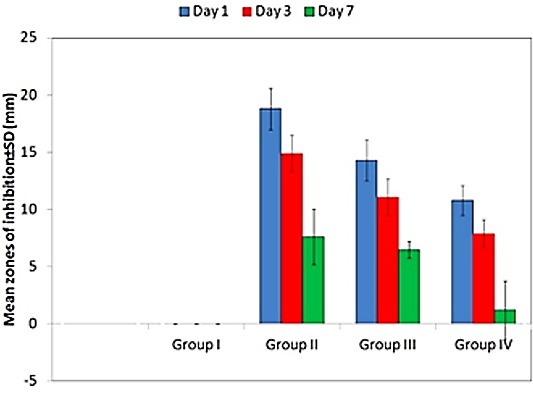
Intragroup wise representation of zones of inhibition

Group I showed no antifungal activity throughout the tested time period of seven days. Figure 1 shows intragroup wise graphical representation of zones of inhibition. Table 1 illustrates the intergroup comparison.

**Table 1:** Intergroup comparison of Zones of inhibition

**Comparison**	**T1**			**T2**			**T3**		
	**MD**	**SE**	**‘p-value’**	**MD**	**SE**	**‘p-value’**	**MD**	**SE**	**‘p-value’**
**I v/s II**	-18.80	0.64	<0.001	-14.90	0.57	<0.001	-7.60	0.80	<0.001
**I v/s III**	-14.30	0.64	<0.001	-11.10	0.57	<0.001	-6.50	0.80	<0.001
**I v/s IV**	-10.80	0.64	<0.001	-7.90	0.57	<0.001	-1.20	0.80	0.445
**II v/s III**	4.50	0.64	<0.001	3.80	0.57	<0.001	1.10	0.80	0.520
**II v/s IV**	8.00	0.64	<0.001	7.00	0.57	<0.001	6.40	0.80	<0.001
**III v/s IV**	3.50	0.64	<0.001	3.20	0.57	<0.001	5.30	0.80	<0.001

MD=Mean difference; SE=Standard error; ‘p-value’=level of significance

### Tensile Bond Strength

**Figure 2:**
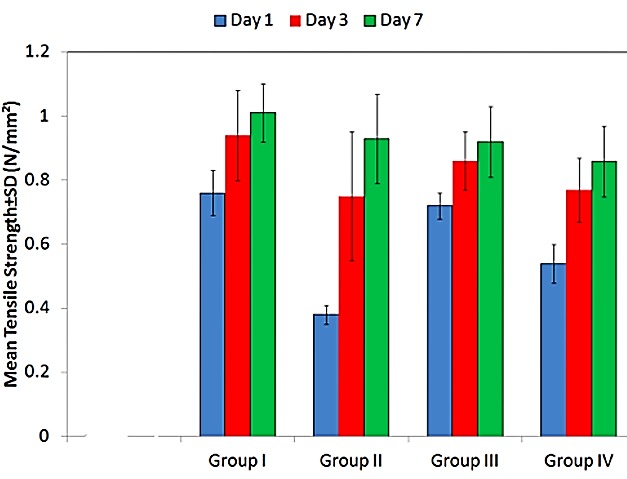
Intra-group representation of tensile bond strength

An increasing trend in tensile bond strength was noticed in all the groups at all time intervals. Intragroup comparison of tensile bond strength is depicted graphically in Figure 2. Intergroup comparison of tensile bond strength is given in Table 2.

### Viscoelasticity

**Figure 3:**
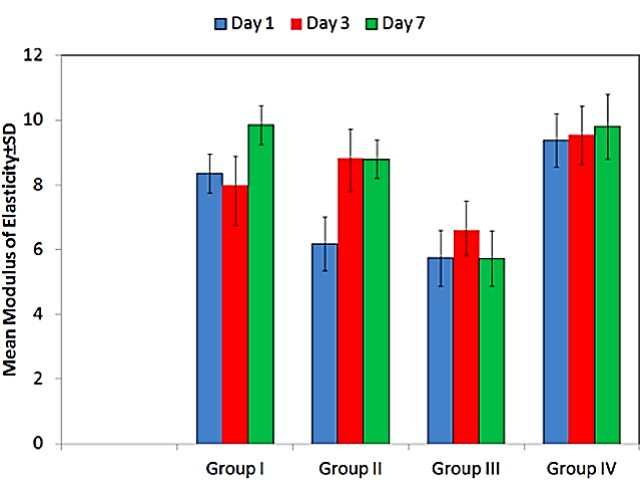
Intra-group representation of modulus of elasticity

Viscoelasticity of the tissue conditioner having different antifungal agents in various concentrations was tested through measurement of modulus of elasticity through stress relaxation test. The tested groups showed varying results at different time intervals. Figure 3 depicts intra-group representation of modulus of elasticity graphically. Table 3 illustrates intergroup comparison of modulus of elasticity.

**Table 2:** Intergroup comparison of tensile bond strength

**Comparison**	**24 hours**			**Day 3**			**Day 7**		
	**MD**	**SE**	**‘p-value’**	**MD**	**SE**	**‘p-value’**	**MD**	**SE**	**‘p-value’**
**I v/s II**	0.39	0.02	<0.001	0.19	0.06	0.018	0.08	0.05	0.408
**I v/s III**	0.04	0.02	0.245	0.08	0.06	0.597	0.09	0.05	0.298
**I v/s IV**	0.22	0.02	<0.001	0.17	0.06	0.047	0.15	0.05	0.038
**II v/s III**	-0.34	0.02	<0.001	-0.12	0.06	0.267	0.01	0.05	0.997
**II v/s IV**	-0.16	0.02	<0.001	-0.02	0.06	0.980	0.06	0.05	0.606
**III v/s IV**	0.18	0.02	<0.001	0.09	0.06	0.469	0.05	0.05	0.735

MD=Mean difference; SE=Standard error; ‘p-value’=level of significance

## Discussion

### Antifungal Property

In the present study, when antifungal property was evaluated, it showed a statistically significant decrease during the period of study. This decrease in zones of inhibition at the end of seven days was due to the regrowth of the fungus.^10^ Similar decreasing trends in the antifungal activity have also been seen in the studies conducted by Sharma et al,^6^ Chopde et al^19^ and Falah-tafti et al^20^ for fluconazole and Srivastava et al^11^ for oregano oil. Although, there are studies confirming the antifungal efficacy of virgin coconut oil as conducted by Ogbolu et al^12^ and Kannan et al,^13^ studies related to decreasing trend in antifungal activity over a period of time have not been previously reported. Although, the mechanism of action of all the three anti-fungal agents is different from each other and all of them are causing destruction of the fungus cell membrane in one way or the other such as, fluconazole acts by inhibiting the fungal cytochrome P450 enzyme 'lanosterol l4-demethylase' causing impairment in ergosterol synthesis leading to a cascade of membrane abnormalities in the fungus;^8^ origanum oil directly inhibits germination and filament formation (the two phases required for tissue invasion) by Candida albicans^21^ and virgin coconut oil acts by causing the cell membrane of the fungus to disintegrate^13^ but still, fluconazole showed highest antifungal activity amongst all. This is because fluconazole is more potent antifungal agent than the oregano oil and virgin coconut oil which could be supported by the fact that the minimum inhibitory concentration of fluconazole against Candida albicans being reported is 0.25-64 mg/ml,^22^ for oregano oil it is 2.5 mg/ml^23^ and for virgin coconut oil it is 32 mg/ml.^12^

**Table 3:** Intergroup comparison of Modulus of Elasticity

**Comparison**	**24 hours**			**Day 3**			**Day 7**		
	**MD**	**SE**	**‘p-value’**	**MD**	**SE**	**‘p-value’**	**MD**	**SE**	**‘p-value’**
**I v/s II**	2.18	0.35	<0.001	-0.84	0.44	0.242	1.06	0.35	0.022
**I v/s III**	2.62	0.35	<0.001	1.38	0.44	0.018	4.14	0.35	<0.001
**I v/s IV**	-1.03	0.35	0.029	-1.57	0.44	0.006	0.06	0.35	0.998
**II v/s III**	0.44	0.35	0.604	2.22	0.44	<0.001	3.08	0.35	<0.001
**II v/s IV**	-3.21	0.35	<0.001	-0.72	0.44	0.374	-0.99	0.35	0.034
**III v/s IV**	-3.64	0.35	<0.001	-2.94	0.44	<0.001	-4.08	0.35	<0.001

MD=Mean difference; SE=Standard error; ‘p-value’=level of significance

### Tensile bond strength

Tensile bond strength showed an increasing trend from day one to day seven in all the groups at all time intervals. These results were in accordance with the study conducted by Huddar et al,^24^ and Yanikogtlu et.^25^ The authors were of the opinion that increase in the bond strength could have occurred because of the leaching out of the plasticizer, which resulted in increased stiffness.This in turn resulted in mechanical bonding and chemical adhesion between soft liner material and acrylic resin.^17,25^ According to Srivastava et al,^11^ the bond strength of Viscogel alone and in combination with oregano oil after one day was 3.97 ±0.75 MPa and 3.73±0.65 MPa which increased to 5.40 ±0.49 MPa and 5.11 ±0.66 MPa respectively after one week. These results were contradictory to the results of the studies conducted by Mese et al,^26^ in which the tensile bond strength of the acrylic resin-based liner (Coe-Soft) decreased over the tested time period from 0.45 MPa after 24 hours to 0.39 MPa after one week because of the swelling and stress formation at the bond interface or from a change in the viscoelastic properties of the liner rendering the material stiffer and better able to transmit external loads to the bond site. On intergroup comparison a statistically significant difference in tensile bond strength was seen after day one (p<0.001) and day three (p=0.014), but, no statistically significant difference was noted after day seven (p=0.060). However, in another study conducted by Srivastava et al no significant difference was seen in tensile bond strength after addition of oregano oil after day one and day seven which indicated that the tensile bond strength of Viscogel was not affected by the addition of oregano oil.^11^ Although, antifungal activity of virgin coconut oil has been previously documented by various authors such as Ogbolu et al^12^and Kannan et al,^13^ its effect on tensile bond strength of tissue conditioner after getting incorporated into it has not been studied as yet.

### Viscoelasticity 

On intragroup comparison, statistically significant difference was found in Group I (p<0.001), Group II (p<0.001) and Group III (p=0.038), while Group IV showed no statistically significant difference (p=0.596). A higher value of modulus of elasticity which was noted at the end of seventh days in all the groups except Group III denoted increased stiffness of material indicating that the viscoelasticity deteriorated with time. This was in accordance with the studies conducted by Saitoh et al,^27^ Mc carthy et al,^18^ and Duran et al^28^ in which the authors concluded that an increase in the elastic modulus was noted over the tested time period rendering the material stiffer. This change in the viscoelasticity resulted from leaching out of the components contained in the liquid, especially ethyl alcohol which are replaced by water.^3,29^ On application of Tukey HSD tests, it was seen that statistically significant increase in elastic modulus was seen for Group I for all time intervals except for day one versus day three (p=0.596). This indicated that Viscogel without any antifungal agents got stiffer at the end of seven days and should be replaced after that. For Group II, this increase in elastic modulus was statistically significant for all time intervals except for day three versus day seven (p=0.999) indicating that the viscoelasticity of Viscogel containing 10% w/w fluconazole deteriorated at the end of three days only and so it should not be used beyond that. For Group III and Group IV the change in elastic modulus was not statistically significant for all time intervals (p>0.05). This implicated that tissue conditioner containing 60%v/v oregano oil (Group III) and 25%v/v virgin coconut oil (Group IV) remained viscoelastic even at the end of seven days and hence, can be used for a longer duration of time. However, studies related to viscoelasticity of the tissue conditioner after addition of fluconazole, oregano oil and virgin coconut oil have not been conducted till now.

The clinical implications of the results of the present study is that the incorporation of various antifungal agents in the tissue conditioner can serve as an alternative to systemic or topical delivery systems of antifungal drug delivery. When mechanical and physical properties were evaluated, oregano oil showed best results amongst all the tested groups. Since, this herbal product have an added advantage of being safe and cost effective, it can be used as an alternative to the synthetic agents that are currently in use.

There are certain limitations in the present study. The present study was aimed at determining the antifungal activity of different antifungal agents incorporated in the tissue conditioner through measurement of zones of inhibition. However, the antifungal potency is also dependent upon the rate of diffusion of these antifungal agents from Viscogel into agar which in turn is affected by it’s concentration, molecular size, viscosity and phase (liquid/solid) of the medium which were not evaluated in the present study.^7^ Therefore, studies based on these parameters affecting diffusion rate should be used to evaluate antifungal potency of these antifungal agents. Since, the present study was performed under controlled laboratory conditions therefore, in-vivo studies are suggested for more precise results.

## Conclusion

Within the limitations of the study, following conclusions were drawn:

- Maximum antifungal activity was shown by Group II followed by Group III and Group IV.
- Group I had the maximum and Group IV had the minimum tensile bond strength at the end of seven days.
- Viscoelasticity deteriorated for Group I and Group II at the end of seven and three days respectively. While, for Group III and Group IV no significant changes were observed for all time intervals.

## Ethical Issues

Not applicable.

## Conflict of Interest

Authors declare no conflict of interest in this study.
